# 2,2′-(Butane-1,4-di­yl)diisoquinolinium tetra­chloridozincate(II)

**DOI:** 10.1107/S1600536808038932

**Published:** 2008-11-26

**Authors:** Zhi-Fang Fan, Xin Xiao, Yun-Qian Zhang, Sai-Feng Xue, Zhu Tao

**Affiliations:** a Key Laboratory of Macrocyclic and Supramolecular Chemistry of Guizhou Province, Guizhou University, Guiyang 550025, People’s Republic of China; b Institute of Applied Chemistry, Guizhou University, Guiyang 550025, People’s Republic of China

## Abstract

The crystal of the title compound, (C_22_H_22_N_2_)[ZnCl_4_], consists of 2,2′-(butane-1,4-di­yl)diisoquinolinium organic cations and [ZnCl_4_]^2−^ complex anions. The cation is located across a twofold axis and the Zn^II^ atom of the anion is located on the other twofold axis. The centroid–centroid distance between parallel pyridine rings of neighboring mol­ecules is 3.699 (3) Å, but the face-to-face separation of 3.601 (3) Å suggests there is no significant π–π stacking in the crystal structure.

## Related literature

For general background, see: Day & Arnold (2000 ▶); Day *et al.* (2002 ▶); Freeman *et al.* (1981 ▶); Kim *et al.* (2000 ▶). For a related structure, see: Pan & Xu (2004 ▶).
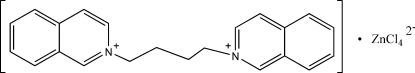


## Experimental

### 

#### Crystal data

(C_22_H_22_N_2_)[ZnCl_4_]
*M* *_r_* = 521.61
Monoclinic, 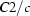

*a* = 10.729 (3) Å
*b* = 11.040 (3) Å
*c* = 18.955 (4) Å
β = 99.179 (9)°
*V* = 2216.4 (10) Å^3^
*Z* = 4
Mo *K*α radiation
μ = 1.60 mm^−1^
*T* = 273 (2) K
0.23 × 0.19 × 0.17 mm

#### Data collection

Bruker SMART CCD area-detector diffractometer
Absorption correction: multi-scan (*SADABS*; Bruker, 2005 ▶) *T*_min_ = 0.680, *T*_max_ = 0.760
12088 measured reflections
2172 independent reflections
1855 reflections with *I* > 2σ(*I*)
*R*_int_ = 0.032

#### Refinement

*R*[*F* ^2^ > 2σ(*F* ^2^)] = 0.058
*wR*(*F* ^2^) = 0.206
*S* = 1.13
2172 reflections
132 parameters
H-atom parameters constrained
Δρ_max_ = 1.28 e Å^−3^
Δρ_min_ = −1.15 e Å^−3^

### 

Data collection: *SMART* (Bruker, 2002 ▶); cell refinement: *SAINT* (Bruker, 2002 ▶); data reduction: *SAINT*; program(s) used to solve structure: *SHELXS97* (Sheldrick, 2008 ▶); program(s) used to refine structure: *SHELXL97* (Sheldrick, 2008 ▶); molecular graphics: *ORTEP-3 for Windows* (Farrugia, 1997 ▶); software used to prepare material for publication: *WinGX* (Farrugia, 1999 ▶).

## Supplementary Material

Crystal structure: contains datablocks global, I. DOI: 10.1107/S1600536808038932/xu2462sup1.cif

Structure factors: contains datablocks I. DOI: 10.1107/S1600536808038932/xu2462Isup2.hkl

Additional supplementary materials:  crystallographic information; 3D view; checkCIF report

## Figures and Tables

**Fig. 1.**
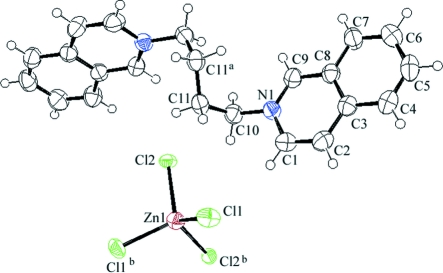
The molecular structure showing the atom-labeling scheme. Displacement ellipsoids are drawn at the 50% probability level [symmetry codes: (a) -x, y, 3/2-z; (b) -x, y, 1/2-z].

## supplementary crystallographic information

### Comment

As part of our ongoing investigation on quinoline compounds, we present here the crystal structure of the compound with multiple functional groups, which can develop strong intermolecular interactions with cucurbit[*n*]urils (CB[*n*]) (Freeman *et al.*, 1981; Day & Arnold, 2000; Day *et al.*, 2002; Kim *et al.*, 2000).

The crystal structure of the title compound (Fig. 1) consists of organic cations and anionic (ZnCl_4_)^2-^ complexes. The (ZnCl_4_)^2-^ anion assumes a distorted tetrahedron coordination geometry with Zn–Cl bond distances of 2.3043 (14) Å and 2.3158 (12) Å. The centroids distance between parallel pyridine rings of neighboring molecules is 3.699 (3) Å, but the face-to-face separation of 3.601 (3) Å suggests no significant π-π stacking in the crystal structure (Pan & Xu, 2004).

### Experimental

A solution of 1,4-dibromine-butane (2.16 g, 0.01 mol) was added to a stirred solution of isoquinoline (2.58 g, 0.02 mol) in 1,4-dioxane (50 ml) at 373 K in a period of 5 h. After cooling to room temperature, the mixture was filtered. The residue was added to an aqueous solution (50 ml) of ZnCl_2_ (0.01 mol, 1.37 g). After stirring for 2 h, the solution was filtered. Colorless single crystals of the title compound were obtained from the filtrate after 5 weeks.

### Refinement

H atoms were placed in calculated positions with C—H = 0.93 (aromatic) or 0.97 Å (methylene), and refined in riding mode with *U*_iso_(H) = 1.2*U*_eq_(C). The highest peak and deepest hole in the final d-map are 0.35 Å from Cl2 atom and 0.42 Å from Zn1 atom, respectively.

### Crystal data

**Table:**

(C_22_H_22_N_2_)[ZnCl_4_]	*F*_000_ = 1064
*M**_r_* = 521.61	*D*_x_ = 1.563 Mg m^−^^3^
Monoclinic, *C*2/*c*	Mo *K*α radiation λ = 0.71073 Å
Hall symbol: -C 2yc	Cell parameters from 2184 reflections
*a* = 10.729 (3) Å	θ = 2.2–26.0º
*b* = 11.040 (3) Å	µ = 1.60 mm^−^^1^
*c* = 18.955 (4) Å	*T* = 273 (2) K
β = 99.179 (9)º	Prism, colorless
*V* = 2216.4 (10) Å^3^	0.23 × 0.19 × 0.17 mm
*Z* = 4	

### Data collection

**Table:**

Bruker SMART CCD area-detector diffractometer	2172 independent reflections
Radiation source: fine-focus sealed tube	1855 reflections with *I* > 2σ(*I*)
Monochromator: graphite	*R*_int_ = 0.032
*T* = 273(2) K	θ_max_ = 26.0º
φ and ω scans	θ_min_ = 2.2º
Absorption correction: multi-scan(SADABS; Bruker, 2005)	*h* = −13→13
*T*_min_ = 0.680, *T*_max_ = 0.760	*k* = −13→13
12088 measured reflections	*l* = −23→21

### Refinement

**Table:**

Refinement on *F*^2^	Secondary atom site location: difference Fourier map
Least-squares matrix: full	Hydrogen site location: inferred from neighbouring sites
*R*[*F*^2^ > 2σ(*F*^2^)] = 0.058	H-atom parameters constrained
*wR*(*F*^2^) = 0.206	*w* = 1/[σ^2^(*F*_o_^2^) + (0.1169*P*)^2^ + 12.0521*P*] where *P* = (*F*_o_^2^ + 2*F*_c_^2^)/3
*S* = 1.13	(Δ/σ)_max_ < 0.001
2172 reflections	Δρ_max_ = 1.28 e Å^−^^3^
132 parameters	Δρ_min_ = −1.15 e Å^−^^3^
Primary atom site location: structure-invariant direct methods	Extinction correction: none

### Special details

**Table:**

Geometry. All e.s.d.'s (except the e.s.d. in the dihedral angle between two l.s. planes) are estimated using the full covariance matrix. The cell e.s.d.'s are taken into account individually in the estimation of e.s.d.'s in distances, angles and torsion angles; correlations between e.s.d.'s in cell parameters are only used when they are defined by crystal symmetry. An approximate (isotropic) treatment of cell e.s.d.'s is used for estimating e.s.d.'s involving l.s. planes.
Refinement. Refinement of *F*^2^ against ALL reflections. The weighted *R*-factor *wR* and goodness of fit *S* are based on *F*^2^, conventional *R*-factors *R* are based on *F*, with *F* set to zero for negative *F*^2^. The threshold expression of *F*^2^ > σ(*F*^2^) is used only for calculating *R*-factors(gt) *etc*. and is not relevant to the choice of reflections for refinement. *R*-factors based on *F*^2^ are statistically about twice as large as those based on *F*, and *R*- factors based on ALL data will be even larger.

### Fractional atomic coordinates and isotropic or equivalent isotropic displacement parameters (Å^2^)

**Table:**

	*x*	*y*	*z*	*U*_iso_*/*U*_eq_	
Zn1	0.0000	0.15361 (8)	0.2500	0.0412 (3)	
Cl2	0.12762 (10)	0.03371 (11)	0.19106 (6)	0.0349 (4)	
Cl1	0.12438 (13)	0.27560 (13)	0.33123 (8)	0.0483 (4)	
N1	0.4868 (4)	0.1136 (4)	0.3832 (2)	0.0416 (10)	
C11	0.4355 (5)	0.1981 (5)	0.2617 (3)	0.0449 (13)	
H11A	0.4248	0.2744	0.2854	0.054*	
H11B	0.3715	0.1945	0.2193	0.054*	
C3	0.6202 (6)	0.1461 (5)	0.5206 (3)	0.0439 (13)	
C9	0.5975 (6)	0.0584 (5)	0.4013 (3)	0.0442 (13)	
H9	0.6286	0.0098	0.3680	0.053*	
C10	0.4120 (6)	0.0967 (6)	0.3108 (3)	0.0463 (13)	
H10A	0.3228	0.0938	0.3143	0.056*	
H10B	0.4347	0.0203	0.2910	0.056*	
C1	0.4382 (6)	0.1876 (6)	0.4311 (3)	0.0528 (15)	
H1	0.3614	0.2265	0.4171	0.063*	
C8	0.6687 (5)	0.0723 (5)	0.4702 (3)	0.0415 (12)	
C6	0.8489 (7)	0.0222 (6)	0.5569 (4)	0.0583 (16)	
H6	0.9255	−0.0177	0.5700	0.070*	
C7	0.7856 (6)	0.0107 (6)	0.4893 (3)	0.0533 (15)	
H7	0.8182	−0.0368	0.4560	0.064*	
C5	0.7995 (7)	0.0947 (6)	0.6080 (3)	0.0581 (17)	
H5	0.8434	0.1003	0.6543	0.070*	
C4	0.6894 (7)	0.1560 (6)	0.5902 (3)	0.0524 (15)	
H4	0.6594	0.2046	0.6239	0.063*	
C2	0.5011 (7)	0.2039 (6)	0.4980 (3)	0.0542 (15)	
H2	0.4668	0.2532	0.5297	0.065*	

### Atomic displacement parameters (Å^2^)

**Table:**

	*U*^11^	*U*^22^	*U*^33^	*U*^12^	*U*^13^	*U*^23^
Zn1	0.0383 (5)	0.0406 (6)	0.0448 (6)	0.000	0.0074 (4)	0.000
Cl2	0.0276 (6)	0.0410 (7)	0.0363 (6)	0.0026 (4)	0.0056 (4)	−0.0100 (5)
Cl1	0.0393 (7)	0.0457 (8)	0.0587 (9)	−0.0063 (6)	0.0043 (6)	−0.0239 (6)
N1	0.038 (2)	0.046 (3)	0.040 (2)	0.000 (2)	0.0034 (18)	0.0025 (19)
C11	0.048 (3)	0.045 (3)	0.039 (3)	0.005 (2)	−0.001 (2)	0.002 (2)
C3	0.047 (3)	0.044 (3)	0.041 (3)	−0.005 (2)	0.008 (2)	0.000 (2)
C9	0.050 (3)	0.044 (3)	0.038 (3)	0.001 (2)	0.006 (2)	−0.001 (2)
C10	0.041 (3)	0.053 (3)	0.043 (3)	−0.006 (3)	0.002 (2)	0.000 (2)
C1	0.045 (3)	0.061 (4)	0.054 (4)	0.006 (3)	0.012 (3)	0.003 (3)
C8	0.043 (3)	0.041 (3)	0.040 (3)	−0.003 (2)	0.005 (2)	0.002 (2)
C6	0.052 (4)	0.058 (4)	0.061 (4)	0.005 (3)	−0.001 (3)	0.006 (3)
C7	0.056 (4)	0.056 (4)	0.045 (3)	0.009 (3)	0.000 (3)	0.001 (3)
C5	0.066 (4)	0.062 (4)	0.042 (3)	−0.014 (3)	−0.005 (3)	0.008 (3)
C4	0.066 (4)	0.053 (4)	0.039 (3)	−0.006 (3)	0.009 (3)	−0.002 (2)
C2	0.060 (4)	0.060 (4)	0.045 (3)	0.009 (3)	0.016 (3)	−0.005 (3)

### Geometric parameters (Å, °)

**Table:**

Zn1—Cl1^i^	2.3043 (14)	C9—H9	0.9300
Zn1—Cl1	2.3043 (14)	C10—H10A	0.9700
Zn1—Cl2^i^	2.3158 (12)	C10—H10B	0.9700
Zn1—Cl2	2.3158 (12)	C1—C2	1.350 (9)
N1—C9	1.330 (7)	C1—H1	0.9300
N1—C1	1.384 (8)	C8—C7	1.423 (9)
N1—C10	1.488 (7)	C6—C7	1.357 (9)
C11—C10	1.502 (8)	C6—C5	1.423 (10)
C11—C11^ii^	1.520 (12)	C6—H6	0.9300
C11—H11A	0.9700	C7—H7	0.9300
C11—H11B	0.9700	C5—C4	1.356 (10)
C3—C4	1.412 (9)	C5—H5	0.9300
C3—C8	1.416 (8)	C4—H4	0.9300
C3—C2	1.431 (9)	C2—H2	0.9300
C9—C8	1.411 (8)		
			
Cl1^i^—Zn1—Cl1	108.47 (9)	N1—C10—H10B	109.4
Cl1^i^—Zn1—Cl2^i^	109.41 (5)	C11—C10—H10B	109.4
Cl1—Zn1—Cl2^i^	109.62 (5)	H10A—C10—H10B	108.0
Cl1^i^—Zn1—Cl2	109.62 (5)	C2—C1—N1	120.7 (6)
Cl1—Zn1—Cl2	109.41 (5)	C2—C1—H1	119.7
Cl2^i^—Zn1—Cl2	110.28 (7)	N1—C1—H1	119.7
C9—N1—C1	121.0 (5)	C9—C8—C3	118.9 (5)
C9—N1—C10	120.6 (5)	C9—C8—C7	120.6 (5)
C1—N1—C10	118.4 (5)	C3—C8—C7	120.4 (5)
C10—C11—C11^ii^	115.5 (4)	C7—C6—C5	120.7 (6)
C10—C11—H11A	108.4	C7—C6—H6	119.7
C11^ii^—C11—H11A	108.4	C5—C6—H6	119.7
C10—C11—H11B	108.4	C6—C7—C8	119.0 (6)
C11^ii^—C11—H11B	108.4	C6—C7—H7	120.5
H11A—C11—H11B	107.5	C8—C7—H7	120.5
C4—C3—C8	118.7 (6)	C4—C5—C6	121.1 (6)
C4—C3—C2	123.8 (6)	C4—C5—H5	119.5
C8—C3—C2	117.5 (5)	C6—C5—H5	119.5
N1—C9—C8	121.2 (5)	C5—C4—C3	120.1 (6)
N1—C9—H9	119.4	C5—C4—H4	120.0
C8—C9—H9	119.4	C3—C4—H4	120.0
N1—C10—C11	111.1 (5)	C1—C2—C3	120.7 (6)
N1—C10—H10A	109.4	C1—C2—H2	119.7
C11—C10—H10A	109.4	C3—C2—H2	119.7
			
C1—N1—C9—C8	0.7 (9)	C2—C3—C8—C7	−179.1 (6)
C10—N1—C9—C8	−179.1 (5)	C5—C6—C7—C8	0.0 (10)
C9—N1—C10—C11	−96.2 (6)	C9—C8—C7—C6	−177.3 (6)
C1—N1—C10—C11	83.9 (6)	C3—C8—C7—C6	1.0 (9)
C11^ii^—C11—C10—N1	71.4 (7)	C7—C6—C5—C4	−1.3 (10)
C9—N1—C1—C2	−1.2 (9)	C6—C5—C4—C3	1.6 (10)
C10—N1—C1—C2	178.6 (6)	C8—C3—C4—C5	−0.5 (9)
N1—C9—C8—C3	0.3 (8)	C2—C3—C4—C5	177.7 (6)
N1—C9—C8—C7	178.6 (6)	N1—C1—C2—C3	0.7 (10)
C4—C3—C8—C9	177.6 (5)	C4—C3—C2—C1	−178.0 (6)
C2—C3—C8—C9	−0.7 (8)	C8—C3—C2—C1	0.2 (10)
C4—C3—C8—C7	−0.8 (9)		

Symmetry codes: (i) −*x*, *y*, −*z*+1/2; (ii) −*x*+1, *y*, −*z*+1/2.
